# Aging in Persons with Rett Syndrome: An Updated Review

**DOI:** 10.1100/tsw.2010.79

**Published:** 2010-05-04

**Authors:** Meir Lotan, Joav Merrick, Isack Kandel, Mohammed Morad

**Affiliations:** ^1^ Department of Physical Therapy, Ariel University Center of Samaria, Ariel, Israel; ^2^ Israeli Rett Syndrome Association, National Evaluation Team, National Rett Syndrome Clinic Chaim Sheba Medical Center, Ramat-Gan, Israel; ^3^ National Institute of Child Health and Human Development, Office of the Medical Director, Division for Mental Retardation, Ministry of Social Affairs, Jerusalem, Israel; ^4^ Kentucky Children's Hospital, University of Kentucky, Lexington, KY, USA; ^5^ Department of Family Medicine, Faculty of Health Sciences, Ben Gurion University of the Negev, Beer-Sheva, Israel

**Keywords:** Rett syndrome, adults, aging

## Abstract

Rett syndrome (RS) is a neurological disease affecting mainly females, characterized by an arrest of brain development caused by an X-linked mutation. Rett syndrome is the first human disease found to be caused by defects in a protein involved in regulating gene expression through its interaction with methylated DNA. The disease has been traced to a defective gene called MECP2. The case stories presented here and recent findings show that females with RS are able to live into old age. Due to the observed longevity of individuals with RS, and the fact that individuals with RS present the therapist/physician with specific clinical challenges, it is suggested that proper, long-term, and individually tailored, intensive care should be provided at all ages in the hope to prevent or at least reduce the age-related deterioration that is typical of this population.

## INTRODUCTION

Until recently, most individuals with intellectual disabilities (ID) lived much shorter life spans in comparison to individuals without ID. At present, adults with ID are living to advanced ages due to developments in medical care and technology that have increased life expectancy of this population[1,2,3]. An estimated 641,000 adults with ID ages 60 years and up were residing in the U.S. in the year 2000, and official expectations are that this group will increase threefold by 2020[4]. Not only that adults with ID are living longer and healthier lives, and their life expectancies have been found to have increased substantially over the last few decades, expectations are that this trend will continue in future years[5]. As persons with ID are living longer, geriatrics health care providers need to learn about the characteristics, health care needs, and common clinical issues facing this population in general, as well as subgroups within this group of clients, such as persons with Rett syndrome.

## RETT SYNDROME

The original observation of six girls with a peculiar disease, reported by Andreas Rett (1924–1997) from Austria, was published in German in 1966[6], but this syndrome only gained international attention in 1983 when Hagberg and colleagues[7] published their findings on 35 cases. Hagberg had, in fact, already observed his first patient in 1960[8].

Rett syndrome (RS) is a neurological disease[9] affecting mainly females[10], characterized by an arrest of brain development[9] caused by an X chromosome mutation. RS is the first human disease found to be caused by defects in a protein involved in regulating gene expression through its interaction with methylated DNA[11] and has been traced to a defective gene on the X chromosome called MECP2 (pronounced "meck-pea-two"). This discovery was made by Amir, an Israeli physician who found the first mutations[11,12] while working at the laboratory of Zoghbi, a neurogeneticist at Baylor College of Medicine in Houston, Texas.

RS typically presents after the first year of life (6–18 months of age) and is one of the most common causes of multiple disabilities among females. The disease incidence is one in 10,000–15,000 of live female births, with a gradual reduction of speech and purposeful hand use, seizures, autistic-like behavior, ataxia, intermittent hyperventilation, and stereotypic hand movements. After initial regression, the condition stabilizes and patients usually survive into adulthood[8].

Although 50–75% of patients achieve independent mobility in early childhood, about 75% lose the ability to walk in later years and become wheelchair bound[13]. Some professionals recommend walking and/or other physical fitness programs as a preventive intervention that might hold back or diminish secondary regression[13,14,15,16]. The debilitating disabilities common to this syndrome are scoliosis (appearing in up to 85% of affected individuals[14]), constipation, and osteoporosis at a young age. All the above medical conditions have been known to be affected positively by physical therapy and other intervention programs, suggesting physical activity for this population.

## RETT SYNDROME STAGES

The stages of RS have been described as: onset or preregression (or stage 1); destructive, motor deterioration, or regression (or stage 2); essentially stable or plateau (or stage 3); and in some cases the term fourth stage (late motor deterioration) has been used in circumstances in which an individual who achieved walking abilities at a young age becomes very handicapped and loses her ambulation[17,18]. Development in females with RS proceeds in an apparently normal fashion *in utero* and during the first 6–18 months of life, at which point their development comes to a halt with regression and loss of many of their acquired skills[19]. Thereafter, a rapid deterioration, with loss of acquired speech and purposeful hand use, ensues. A deceleration of head growth and jerky body movements of the trunk and limbs accompany the developmental deterioration in individuals with RS. Typically, they present with a broad-based gait and side-to side swaying movements of the shoulders when walking[20]. Other physical problems, such as seizures, scoliosis, and breathing abnormalities may appear[21], which require constant care for the rest of the person's life[22]. The scoliosis is a prominent feature in females with RS, but can vary from mild to severe[23]. Apraxia (developmental dyspraxia), the inability to program the body to perform motor tasks, is the most fundamental and severely handicapping aspect of the syndrome. Apraxia can interfere with all body movements, including eye gaze and speech, making it difficult for individuals with RS to execute what they want to do. To sum up, the child adolescent and adult with RS is in need of an intensive and comprehensive management program throughout their life span (estimated life expectancy of individuals with RS in the past was around 50 years)[24].

## LONGITUDINAL FOLLOW-UP OF ADULTS WITH RETT SYNDROME

Studies conducted on aging and RS are scarce, with only a few case studies available. The present section describes longitudinal follow-up of adults with RS, thereby emphasizing the importance of such documentation. One case study[25] described a woman with RS from Norway, who lived to the age of 60 years. That paper provides no information about any genetic test, only a clinical diagnosis. The study was based on medical records, older and more recent videotapes, and interviews with her sister and care-giving staff. After 21 years without being able to walk, following intensive physiotherapy, the woman regained that ability, walking without support. A few years before she died, she also showed improvement in hand use. During the early regression of ambulation at around the age of 20, which was due to moving from living at home to a residential facility, she appeared to lose social interest. The interest improved after some time, but she remained wary of people whom she did not know.

Another case study was published from Denmark of a 77-year-old woman[26], born in 1923 after a normal pregnancy and delivery, who walked unsupported at the age of about 1. She deviated from normal development at 2 years of age. At 38 years of age, she had lost all purposeful hand use and performed the hand mannerisms typical of RS constantly. She developed severe kyphosis and at 41 years of age, ambulation was lost. At age 66, she was diagnosed as RS with the following mutation in her MECP2 alleles: a C to P transition in exon 4 leading to a substitution of threonine by methionine at position 158, T158M in the conserved methyl binding domain (MBD), of the corresponding gene product. The XCI showed a nonrandom pattern with an inactivation ratio of 10:90. This T158 mutation is a common mutation in RS and her skewed XCI may have resulted in her long survival. She died, at the age of 79, from peritonitis caused by an abscess after the removal of a large spleen.

Hagberg[27] described three cases to illustrate long-term clinical follow-up in RS. The first case study was of a girl born in 1957 who had developmental delay and was referred at 3½ years of age, but not diagnosed with RS until 19 years of age. At that time, the main problems were her apraxia, her general developmental retardation, and her aggressive behavior. In addition, she was considerably growth retarded, and had developed a severe kyphosis and a more modest left convex C scoliosis. Her epileptiform symptomatology, which started at age 6 months, was under full control and no longer a problem. At the last follow-up (age 47 years), she was a very small woman, 134 cm tall, weighing 50 kg, with an occipitofrontal head circumference (OFC) of 54 cm. She had short, thin, slightly distorted feet (only 34 cm long). She was still able to walk unsupported, but showed some balance problems. She was less active in motor terms and had signs of excessively early gross motor-muscle aging of the legs. She had hand-finger stereotypes more or less continuously, but a more subtle form of the regular hand stereotypic wringing and twisting performed by females with RS. Her neurology was characterized by a complex gross motor dysfunction of the RS type. She had a complex MECP2 rearrangement with deletions both of exon 3 and exon 4.

The second case study from Hagberg was of a female born in 1960, the second of three siblings, in a healthy family. Her pre- and perinatal history was uneventful with birth at full term (weight 3,720 g, length 50 cm, OFC 34.5 cm). Her first 1¼ years of life were reported to have been uneventful. At that age, she was able to walk with support, pincer grasp, and manipulated toys as expected. She had, however, never learned to crawl on all fours. At that point, a general stagnation occurred, followed rapidly by marked developmental regression. At the age of 1¾ years, she stopped walking completely, lost contact with her parents, developed intense “hand-clapping” stereotypes (film documented), and showed "autistic-like" behaviors. In parallel, her head growth curve indicated a marked deceleration. At 23 years of age, she was diagnosed as a classic RS patient. At the last visit (44 years of age), she was extremely handicapped, very small and thin, with a small head (height 130 cm, weight 24 kg, OFC 48.5 cm). She had a complex S-curved RS kyphoscoliosis and a markedly distended abdomen of the RS bloating type. Her feet were cold and sweaty, bluish in color, and extremely thin and small (length only 22 cm). Her examination was characterized by a dystonic-rigid syndrome of the advanced RS type with a right-sided dominance. She was reported to have repeated, unmotivated, long laughing attacks, as well as paroxysmal night screaming. She had never had any epileptic seizures. She had a commonly found MECP2 mutation in exon 4 (R270X).

The third case, born in 1965, was one of two siblings in a healthy family. Her peri- and neonatal histories were uneventful, as were her developmental abilities when she was born at full term (weight 3,590 g, length 50 cm, OFC 34 cm). She developed normally, but at a health checkup at 1 year, she was considered to be "late", but able to creep on her knees and sit up and walk with support and play with toys. She could say many single words. After 1½ years of age, she slowly regressed, did not use her hands as before, was found socially detached from her parents, and was more or less in her "own world". At the same time, she stopped crawling and talking, and repeatedly had unmotivated screaming attacks. At the age of 2½ years, her skull growth had stagnated significantly. She was slightly hypotonic and had some sort of ataxic movement patterns of the truncal ataxia type, as well as intention-tremor in her hands and stereotypic movement patterns. She did not develop seizures. Throughout the following 3 decades, she successively deteriorated neurologically into a generalized most severe dystonic-atrophic syndrome, with side-asymmetric secondary deformities, contractures, and a collapsed scoliosis. She died at the age of 36 years in a deformed, growth-retarded, and emaciated state.

## RECENT FINDINGS

A large North American cohort (n = 1,928) examined the longevity of individuals with RS and found that about 80% of individuals diagnosed with atypical RS and 60% of individuals with typical RS survive to the age of 50 years[28]. Nevertheless, the authors themselves suggest that given that clinical management has improved considerably from the time when RS was first recognized, improved survival among each successive cohort might be expected. Moreover, we believe that since the research was done only on people currently diagnosed with RS, institutionalized undiagnosed adults might change the data, suggesting even greater longevity in this population.

In another project conducted by researchers from Maastricht University in Holland in association with the Dutch Rett Syndrome Association, questionnaires were sent to families and caregivers of individuals with RS aged 16 years and older[29]. It was found that of the 53 responses to the questionnaire concerning living conditions and use of care facilities, about a third (36%) of the research population resided with their parents and the rest (71%) in residential facilities.

Health of the individuals was assessed on a 5-point scale ranging from very good (1) to very bad (5). In general, the respondents valued the health of individuals with RS as good (mean 2.15), but a significant relationship was found between health and apnea, breath-holding spells, mood changes, spasticity, and joint deformities. In regards to weight status, 49% of the participants were underweight, 40% had a normal weight, and 11% were overweight. In regards to communication, a third of the participants with RS were able to express themselves sometimes by spoken language and/or signals. Communication was found considerably better in the older age groups. These findings support previous knowledge regarding adults with RS[30,31,32].

Cold feet were noticed in 96% of participants with RS, and pressure sores and vesicles occurred in 46%. Half of the participants with RS showed sleeping problems on a nightly basis and the prevalence of sleeping problems was higher in the older age groups. Daytime sleeping was reported in 85% of the research population. Apnea (38%), hyperventilation (39%), breath-holding spells (73%), and air swallowing (41%) were reported with a much lower prevalence in the oldest age group (in regards to apnea).

Night screaming was reported in 39%, prevalence of mood changes in 66%, and abnormal agitation reported in 54%. Two-thirds of the research population showed anxiety. The prevalence of scoliosis in the study was 90%; of those, 36% had undergone surgery, while the prevalence of kyphosis was found in 16%.

In contrast to other areas, gross motor function in the research population was found to slowly, but continuously, decline over the years, which has also been found by other researchers[17,33,34,35].

Ambulation and mobility were very limited in all age groups and no relationship with age was found. The prevalence of spasticity was 52%, mainly affecting the arms and legs. Joint deformities, mostly of the feet were found in 60% of the research population. A history of epilepsy was present in 74%, of whom 95% used anticonvulsive treatments.

In general, in both research projects, better communication and autonomic function in the oldest age group was found compared to the younger age groups, which is in line with previous findings[35,36,37]. The research demonstrates the potential for prolonged survival in this population and suggests the need for careful planning for long-term care, as well as continued observation of the effects of improved clinical management on longevity.

## CLINICAL MANIFESTATIONS OF THE AGING PERSON WITH RETT SYNDROME

The person with RS presents some clinical manifestations that are common for this syndrome and therefore require specific acknowledgment, proper evaluation, and specific intervention:

- Scoliosis — 80–85% of individuals with RS[30,38] are diagnosed with scoliosis. It has been found that intensive therapeutic intervention with adequate sensory[21] and physical[15] support can regress the deterioration of the scoliosis[15] and might even prevent the child from the need to undergo corrective surgery[21].
- Epilepsy — According to differing studies, 30–90% of individuals with RS will be diagnosed with epilepsy[30,36,39,40,41]. Individuals with RS show acute reactions to antiepileptic medication and, therefore, the diagnostic procedure and medication prescription should be performed by physicians acquainted with this specific disorder[30]. Moreover, since many individuals with RS tend to have irregular sleep at night[42] and excessive amounts of daytime sleep[43], antiepileptic medication should be introduced only if the seizures disturb the child's daily routine or functions. Since it has been found that 82% of individuals with RS show breathing abnormalities that might sometimes appear as epileptic-like attacks, telemetry or electroencephalogram (video EEG) should be performed to avoid antiepileptic overmedication. Another aspect of epilepsy typical of this population is the fact that many of them show reduction in the severity and frequency of epileptic attacks in adulthood. Therefore, a slow reduction of anticonvulsive medication should be performed under the supervision of a neurologist knowledgeable of adults with RS[30]. Phenobarbitone and, to a lesser extent, benzodiazepines display a severe effect on the level of alertness and responsiveness of the client with RS, to a point of sudden pseudomotor deterioration. Therefore, their use should be avoided as much as possible[31].
- Carnitine — Carnitine is a biological enhancer for the transport of fatty acids from the cytosol into the mitochondria during the breakdown of lipids. Reduction in carnitine level might cause muscle weakness, liver insufficiency, neurological problems, and hypotonia. All these behaviors might damage the functional ability of the individual with RS, yet they are masked by the fluctuating nature of the RS manifestation. Since individuals with RS have been found to show low levels of carnitine in the past, and since the supply of carnitine has been found to contribute positively to the height, weight, and motor function of these individuals[44,45], carnitine levels should be evaluated and supplied in cases of deficiency. Moreover, the combination of anticonvulsive medication, especially depakot, with carnitine has been found to be beneficial for this population.
- Constipation — Constipation is common in individuals with RS[43]. It is estimated that 85% of individuals with RS will experience severe constipation at least once in their life[46]. Constipation in this population is derived from a lack of physical activity, low muscle tone, improper diet, medication, scoliosis, and reduced liquid intake[47]. Since all the above aspects contributing to constipation are treatable, their occurrence should be examined prior to the use of laxatives and enemas[48].

- Nutrition — Intestinal problems are present in 74% of individuals with RS[41] and are a significant component of this syndrome. There is evidence that these symptoms worsen with age in connection to their functional/orthopedic situation[23,32] and, therefore, constant and proper evaluation should be a part of the follow-up procedure of the adult with RS. Moreover, moderate to severe malnutrition is present in 85% of individuals with RS[49] and is aggravating with age[36,47]; yet many of the nutritional problems of this population are treatable[48] when properly diagnosed.
- Osteoporosis — Osteoporosis occurs frequently in females with RS and has been reported in very young girls[50,51,52]. Patients with RS have been found with decreased bone mineral density compared to controls[53]. These findings support the need for routine checkups from childhood of bone density and the commencement of physical (such as intensive standing and walking programs[16,54]), nutritional[48], and medical interventions[50] as preventive interventions when the situation necessitates such courses of action[31].
- Dental treatment — Specific dental disorder–related problems have been identified in individuals with RS[55,56]. These problems include gum problems, teeth closure[56], bruxism, high risk of falling and facial trauma[32], teeth damage due to prolonged use of anticonvulsive medication, and reflux[21]. These accumulating problems require the dentist's acknowledged attention and care[57,58].
- Functional improvements — A few studies have demonstrated that proper intervention can improve function of children[16] and adults with RS, up to a point were walking was restored for a woman with RS who had stopped walking 20 years previously[25]. Because of the possible life spans of individuals with RS, it is suggested that proper and intensive care should be provided to clients with this syndrome at all ages in the hope of preventing or at least reducing the age-related deterioration that is typical of this population[59].

All the above symptoms are typical of adults with RS and can be treated with conventional intervention, thereby contributing to the longevity and quality of life of this population. Therefore, longitudinal follow-up for proper evaluational procedures and intervention implementation is needed for this population as it ages.

## IDENTIFYING ADULTS WITH RETT SYNDROME

Due to the fact that RS has been acknowledged by Western medicine only in the past 23 years, after the publication of the first English article on RS by Hagberg and colleagues[7], the majority of adults with RS are misdiagnosed and might therefore lack proper intervention.

In Fig. 1, the prevalence of RS in Israel is presented. It is evident from the graph that many RS adults over the age of 15 years have not been located and diagnosed, and older adults (25 years and up) have been scarcely detected.

Due to the specific medical challenges presented by adults with RS, the authors call for a widespread organized search for this population to detect, diagnose, and implement proper intervention programs adapted for the specific needs of individuals with RS. Initial findings from an ongoing survey to detect undiagnosed adults with RS in residential care centers in Israel suggest that about 6% of all females in residential settings might be diagnosed with RS. Moreover, some of the typical characteristics of RS such as bruxism, bloating, sleep disorders, breathing abnormalities, and sometimes even hand mannerisms (reduced to subtle finger movements as described by Hagberg[30]) are less frequent in adulthood than in childhood within this population. Therefore, detection of these individuals requires a person who is highly experienced in RS.

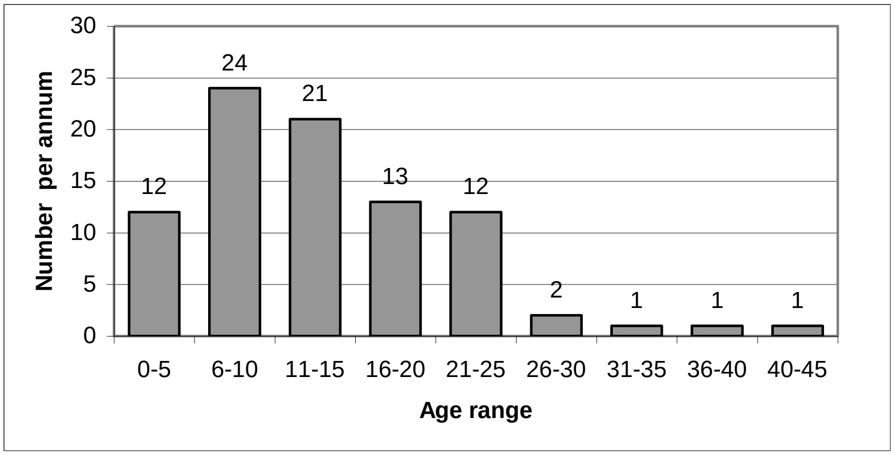

**FIGURE 1.** Prevalence of individuals diagnosed with RS in Israel, according to age.

## MORTALITY IN RETT SYNDROME

People with RS can survive into middle and old age, but life expectancy is reduced and the occurrence of sudden death is greater than in the general population. Longitudinal records of people with RS began in the U.K. in 1982 and developed into the British Survey in 1993. From this British Survey, the mortality rate for RS has been estimated at 1.2% per annum[59], with 48% of deaths occurring in debilitated people, 13% from natural causes, 13% with prior severe seizures, and 26% sudden and unexpected[59,60,61,62]. Respiratory dysrhythmias were usually present. Neuropathological studies confirmed reductions in cortical dendrites and in one case, immaturity of cardiac-conducting tissues[59]. The possible causes of sudden death can include brainstem autonomic failure (respiratory failure, apnea, cardiac arrhythmias)[59,60,61,62].

## DISCUSSION

This review of aging individuals with RS revealed very few studies, and we have still to wait for additional, larger follow-up studies and large cohort studies in the future.

The five case studies from Norway, Denmark, and Sweden showed deceleration of head growth appearing at an early stage, which was associated with increased motor disability and with the appearance of epilepsy[27]. The hand stereotypes, which are the trademark of RS from early childhood, usually change in adult middle age toward frozen and stiff malpositions or remain as finger movement. From childhood to young adulthood, some gross motor functions appear to improve slightly with a temporary recovery and compensation, in contrast to the loss of fine motor abilities. In the long term, middle-aged women with RS lose a great deal of muscle volume, strength, and power, and present with generally premature neuromuscular aging, necessitating the introduction of physical fitness intervention programs. The early general growth deceleration not only affects body and skull growth, but also involves the overly thin, small, cold, and sweaty feet, and the insufficient, compressed, and curved spine[15,27].

Epilepsy occurs in more than 90% of cases with onset of clinical seizures at around 3–5 years of age, with a peak frequency in adolescence, into young adulthood, and successively decreases in early middle age, with only rare and minor problems occurring after the age of 40[27,63]. Therefore, after 40 years of age, an attempt should be made to withdraw antiepileptic drugs gradually[27,63].

Many common characteristics of adults with RS are known. These include constipation, osteoporosis, potential for improvement in functional abilities, dental problems, nutritional needs, orthopedic problems, as well as medication needs. All the above aspects necessitate special evaluation, which should lead to improved, more focused intervention for this population. Nevertheless, the novelty of this syndrome requires a search for undiagnosed adults with RS to enable the implementation of proper care.

The case stories presented here and recent findings show that females with RS can live even to the age of 79 years and suggest significant longevity for this population. Due to the observed longevity of individuals with RS, it is suggested that proper, long-term and intensive care should be provided at all ages in the hope of preventing or at least reducing the age-related deterioration that is typical of this population.
